# Identification of In Vitro Metabolites of Synthetic Phenolic Antioxidants BHT, BHA, and TBHQ by LC-HRMS/MS

**DOI:** 10.3390/ijms21249525

**Published:** 2020-12-15

**Authors:** Ons Ousji, Lekha Sleno

**Affiliations:** Chemistry Department, Université du Québec à Montréal, Downtown Station, P.O. Box 8888, Montréal, QC H3C 3P8, Canada; ons.ousji@gmail.com

**Keywords:** BHT, BHA, synthetic antioxidant, metabolites, in vitro incubations, liquid chromatography, high-resolution tandem mass spectrometry

## Abstract

Butylated hydroxytoluene (BHT) and its analogs, butylated hydroxyanisole (BHA) and tert-butyl-hydroquinone (TBHQ), are widely used synthetic preservatives to inhibit lipid oxidation in the food, cosmetic and pharmaceutical industries. Despite their widespread use, little is known about their human exposure and related biotransformation products. The metabolism of these compounds was investigated using in vitro incubations with human and rat liver fractions. Liquid chromatography coupled to high-resolution tandem mass spectrometry was employed to detect and characterize stable and reactive species formed via oxidative metabolism, as well as phase II conjugates. Several oxidative metabolites have been detected, as well as glutathione, glucuronide, and sulfate conjugates, many of which were not previously reported. A combination of accurate mass measurements, MS/MS fragmentation behavior, and isotope-labeling studies were used to elucidate metabolite structures.

## 1. Introduction

Synthetic phenolic antioxidants were developed in the late 1940s [1]. They have been used in food, pharmaceuticals, cosmetic, and petrochemical industries to increase shelf life and to improve the quality, freshness, taste, and texture of consumer products [2]. They are widely used to trap free radicals and delay lipid oxidation in various products [1]. Despite their widespread use, very little is known about human exposure or environmental emissions, which has led to public concern about their health effects and environmental contamination [3]. Butylated hydroxytoluene (BHT), butylated hydroxyanisole (BHA), and tert-butyl-hydroquinone (TBHQ) are among the most used synthetic phenolic antioxidants [4].

BHT, the most frequently used synthetic phenolic antioxidant, is added to food, pharmaceuticals, and cosmetics, as well as an additive in rubber, plastics, mineral oil, and printing inks [5,6]. Reports on BHT toxicity and side effects have been somewhat contradictory. Some studies have shown positive effects of BHT, such as enhancing the intracellular levels of glutathione and related enzymes in rat [7], protecting against cancer due to its antioxidant activity [8], and having tumor reducing effects [6]. On the other hand, it has been shown to cause renal and hepatic damage in rats, increase liver weight, decrease the activities of several hepatic enzymes and exhibit toxic effects in lung tissue [6]. BHT toxicity has been mainly attributed to its metabolism. For instance, Nagai et al. reported that BHT-quinone, one of the major metabolites of BHT, cleaves DNA strands [9]. Moreover, Kupfer et al. [10] demonstrated lung toxicity and tumor production caused by hydroxylated metabolites of BHT.

BHA, a close analog of BHT, is usually found as an isomeric mixture, containing the major 3-*tert*-butyl-4-hydroxyanisole (3-BHA) (90%) and the minor 2-*tert*-butyl-4-hydroxyanisole (2-BHA) (10%) [11]. It has been used as a food additive since the late 1950s [12]. It is also used in animal feed, cosmetics, pharmaceuticals, rubber, biodiesel, and petroleum products [13]. Some studies on BHA have revealed beneficial effects, with anti-tumor and nephroprotective potential [14,15]. Nevertheless, BHA has been shown to be an endocrine disrupter and a carcinogen in rats [16,17]. It can also perturb adipogenesis and increase the incidence of obesity [11,17]. The effects of BHT and BHA on various organs (liver, lung, kidney, blood system, and reproductive system) have been reviewed [18].

TBHQ is also added to a wide range of foods, such as unsaturated vegetable oils, residual frying oils, lard, and infant formula, as well as in biodiesel [19,20]. Several studies documented the chemoprotective effects of TBHQ. However, other in vitro studies, indicated that it can cause DNA damage and in vivo tests suggest it may be carcinogenic, cytotoxic, and genotoxic [19,21]. TBHQ is a major metabolite of BHA resulting from a demethylation reaction [22], and it has been suggested to contribute to the carcinogenicity of BHA [19].

Despite the large number of scientific papers describing the controversial effects of BHT and its analogs using in vitro and in vivo animal models, only very few studies have described these synthetic antioxidants in humans [1,5,23,24]. BHT and BHA have been banned in Japan since 1958 and have use restrictions in infant formulas in the UK. [25] However, BHT, BHA, and TBHQ are currently allowed in Canada, United States, Korea, and certain countries within the European Union [1,3,26,27]. They are categorized as “generally recognized as safe” (GRAS) by the U.S. Food and Drug Administration (FDA) and safe to use in cosmetics by the Cosmetic Ingredient Review (CIR) Expert Panel [1,2,6,26], while the International Agency for Research on Cancer (IARC) classifies BHT as non-carcinogenic (based on limited evidence), BHA as reasonably anticipated to be a human carcinogen and TBHQ as not carcinogenic [13,16,27].

The metabolism of BHT and analogs has been studied by gas chromatography coupled to mass spectrometry (GC-MS) [1,24,28], and by high-performance liquid chromatography (HPLC) [6]. The tissue distribution, excretion, and metabolism of BHT in mice were recently investigated using HPLC-MS/MS and GC-MS [29].

The potential for human exposure of BHT and its analogs, as well as the lack of information surrounding this exposure, support the need to study their biotransformation reactions, especially concerning the formation of reactive metabolites.

In this study, the metabolism of BHT, BHA, and TBHQ was evaluated using liver microsomes and subcellular (S9) fractions from humans and rats. Oxidative metabolites, glutathione (GSH) adducts, glucuronides, and sulfate conjugates have been characterized by liquid chromatography coupled to a quadrupole-time-of-flight high-resolution tandem mass spectrometer. Isotope-labeled BHT analogs were employed to aid in the structural elucidation of metabolites. Two metabolites of BHT, DBP, and BHT-acid, were also purchased to confirm their presence in BHT incubations. The metabolism of these two compounds was also studied to confirm metabolic pathways.

## 2. Results and Discussion

### 2.1. Metabolism of BHT

**Figure 1:**
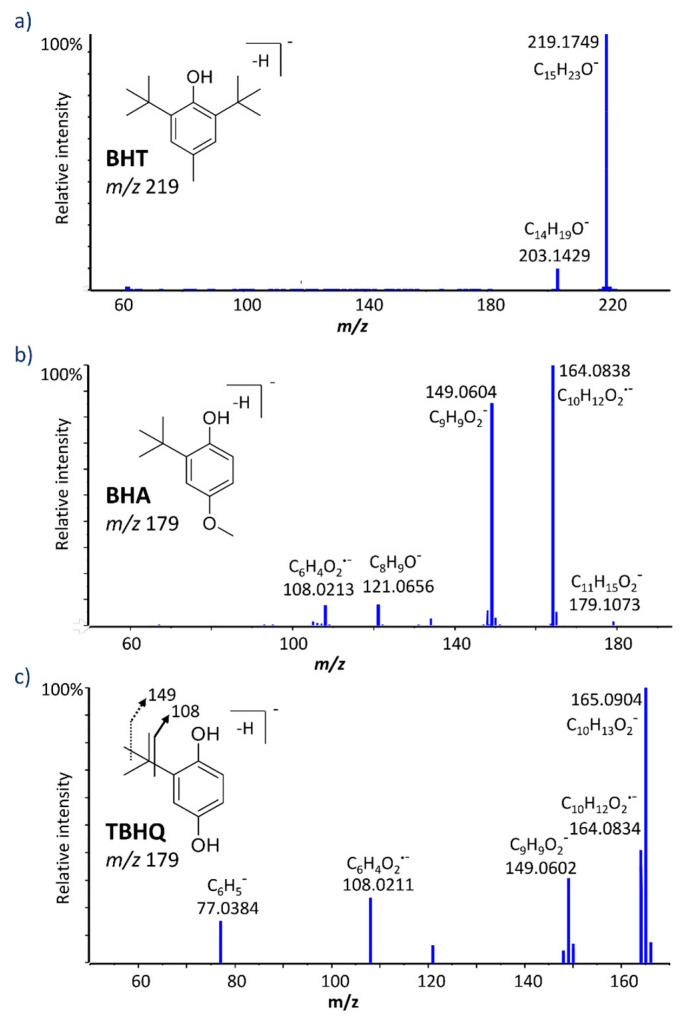
High resolution MS/MS spectra for deprotonated butylated hydroxytoluene (BHT) (**a**), butylated hydroxyanisole (BHA) (**b**), and tert-butyl-hydroquinone (TBHQ) (**c**).

For the purpose of studying the various metabolic routes of BHT, an LC-MS/MS method was optimized. A biphenyl solid-core column using 5 mM ammonium acetate and acetonitrile as mobile phases A and B, respectively, yielded significant signal increase over using formic acid as an additive, as well as ameliorating peak shapes for many of the metabolites found in this study. BHT eluted with a retention time of 14.3 min with our optimized gradient (Table 1). The high-resolution MS/MS spectrum of deprotonated BHT (*m/z* 219.1760) with a collision energy of 30 V, presented in Figure 1, exhibits only one major fragment ion at *m/z* 203.1429 (C_14_H_19_O^−^), via the loss of CH_4_. Otherwise, this molecule is quite resistant to fragmentation, and when collision energy was increased to form other structurally characteristic fragments, most of the signal was lost and no clear product ions were observed.

**Figure 2:**
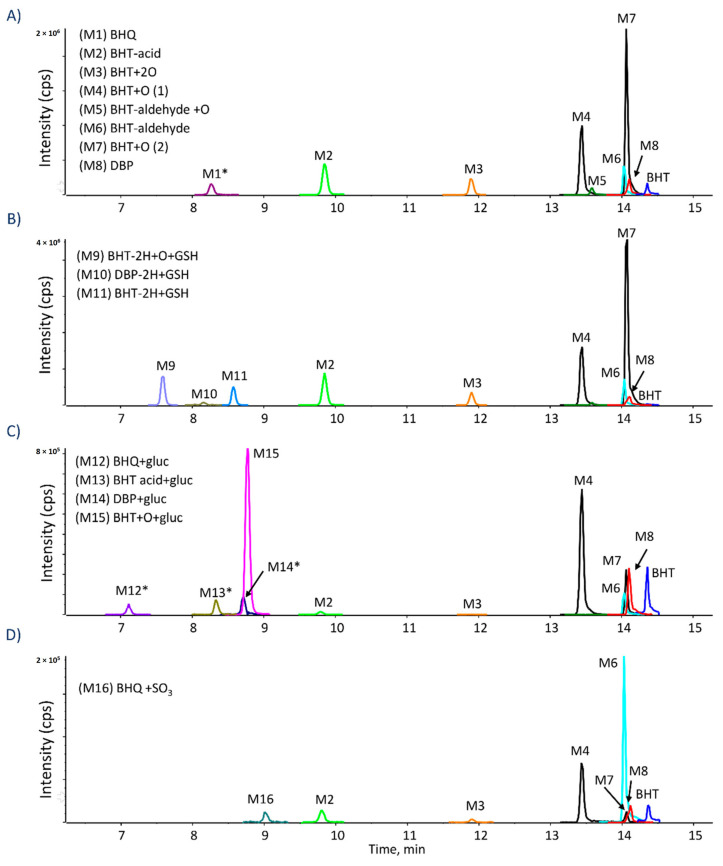
Extracted ion chromatograms of BHT metabolites formed in (**A**) HLM with NADPH; (**B**) HLM with NADPH and GSH; (**C**) HLM with NADPH and UDPGA; (**D**) Human S9 fraction with NADPH and PAPS; Peaks with asterisk. * were increased by 10×.

Both human and rat microsomes yielded similar metabolic profiles for all tested compounds and incubations conditions. Throughout the manuscript, representative chromatograms from the human incubations are shown. All metabolites were confirmed with accurate mass measurements within 5 ppm of the theoretical exact masses. When BHT was incubated under oxidative conditions, several metabolites were detected, as shown in Figure 2. Two hydroxylated BHT metabolites (BHT + O) were detected, eluting at 13.4 and 14.1 min (Table 1). The comparison of their HRMS/MS spectra showed a common water loss at *m/z* 217.159 confirming that, in both cases, the oxygen is not added to the aromatic ring. To investigate whether the oxygen is added to the *para*-methyl or on a *t*-butyl group, isotope-labeled BHTs were also incubated (Table 2). Using BHT-d_3_ and BHT-d_20_, the BHT + O peak at 13.4 min, was shifted from *m/z* 235.1708 to 237.1834 and 255.2967, respectively (Table 2), indicating that the oxidation occurs on the methyl group. While for the isomer at 14.1 min, it was shifted from *m/z* 235.1705 to 238.1893 and 254.2903, respectively (Table 2), proving that the oxidation occurs on a *t*-butyl group. Di-hydroxylated BHT was also detected at 11.9 min (Figure 2) with *m/z* 251.1660 (C_15_H_23_O_3_**^−^**, 2.9 ppm), and its MS/MS spectrum showed two water losses (Table 1). Both BHT-d_3_ and BHT-d_20_ analogs lost one deuterium atom, therefore one oxygen is added on the methyl group and the second one is on the *t*-butyl, in accordance with the two BHT + O isomers mentioned above. BHT-aldehyde via the oxidation of the *p*-methyl group was also detected at 14.0 min (Table 1) [6], confirmed by the fact that BHT-d_3_ lost all three labels during this metabolic transformation, while BHT-d_20_ did not lose any.

Another two oxidative metabolites were detected at 9.8 and 13.5 min, both corresponding to C_15_H_21_O_3_^−^ (*m/z* 249.150). The isomer eluting at 9.8 min showed a characteristic fragment at *m*/*z* 205.1592 corresponding to the loss of CO_2_ (Table 1), and lost the three labels from BHT-d_3_ (Table 2), proving it to be BHT-acid, where the methyl group is oxidized to a carboxylic acid. To further confirm this, the commercial standard of BHT−acid was purchased and showed the same retention time (Figure S1) and MS/MS fragmentation behavior. The second isomer (Table 1), at 13.5 min, presented a water loss consistent with an oxidation on a methyl group, as well as the loss of CH_2_O. The analogous metabolite from BHT-d_20_ had lost one deuterium (*m/z* 268.2689), while BHT-d_3_ lost its three labels (*m/z *249.1504). Taken together, these results confirm this isomer as hydroxylated BHT−aldehyde (BHT−aldehyde + O).

DBP (2,6-di-tert-butylphenol) is a known metabolite of BHT. It is also used as a synthetic phenolic antioxidant in plastics and food packaging [30]. DBP was detected in oxidative incubations at 14.2 min with m/z 205.1600 (Table 1 and Figure 2). This metabolite has been confirmed by the synthetic DBP standard, which was also incubated under oxidative conditions (Figure S1). Further oxidation of DBP can form BHT-hydroquinone (BHQ), which was also detected in BHT incubations at 8.3 min (Table 1 and Figure 2). The loss of all three deuterium labels from BHT-d_3_ and none from BHT-d_20_ confirm the *para*-hydroquinone structure (Table 2) of BHQ.

**Figure 3:**
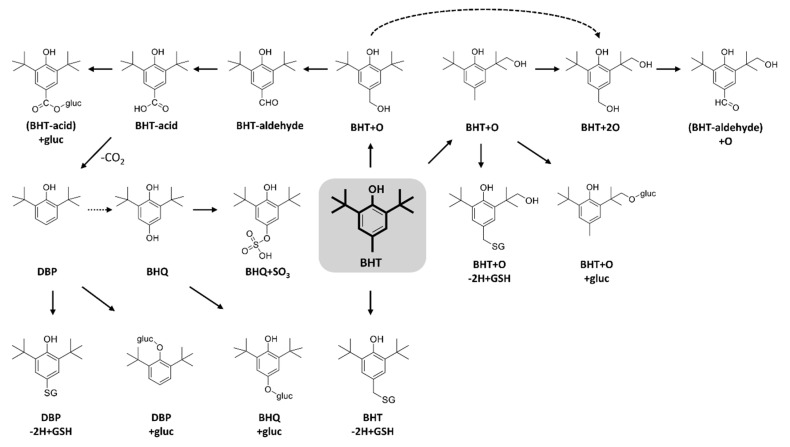
Proposed biotransformation reactions of BHT.

Numerous metabolites of BHT have been described and metabolic pathways proposed [6,31]. A major metabolic pathway is initiated by the oxidation of the *p*-methyl group, leading to the formation BHT-aldehyde and BHT-acid by stepwise oxidation. The acidic form is decarboxylated to DBP, from which BHQ is likely formed. Another metabolic pathway is initiated by the oxidation of the *t*-butyl group. The double oxidation forms BHT + 2O, being further oxidized into hydroxylated BHT-aldehyde (BHT-aldehyde + O) (Figure 3). Thompson et al. [32] studied the oxidative metabolism of BHT by hepatic and pulmonary rodent microsomes and identified several of these metabolites by LC-UV, GC-MS and using radiolabeled BHT (^14^C-BHT). The oxidative pathways of BHT have also been studied in vivo in different species, including the oxidation of the *p*-methyl group as a major metabolic route in rat, rabbit, and monkey, while the oxidation of *t*-butyl groups has been described as the predominant pathway in human and mouse [33]. Zhang et al. [29] reported that BHT and its metabolites, including BHT-acid, BHQ, and BHT-aldehyde were present in metabolism-related organs (e.g., liver and kidney) in mouse.

When GSH was added under oxidative conditions, three adducts were detected (Figure 2), corresponding to BHT-2H + GSH at 8.5 min, BHT + O-2H + GSH at 7.6 min, and DBP-2H + GSH at 8.2 min. HRMS/MS spectra for these adducts were dominated by characteristic peaks from the GSH moiety (m/z 306.077, 272.089, 254.078, 160.007, 143.046, and 128.035) (Table 1). The most prominent fragment ion for BHT-2H + GSH and BHT + O-2H + GSH was m/z 306.07 (Table 1), corresponding to deprotonated GSH. These two metabolites are proposed to result from the formation of the quinone methide intermediate followed by the addition of GSH on the methylene carbon. This hypothesis is supported by results from incubations with isotope-labeled BHT analogs. For BHT-2H + GSH, BHT-d_3_ lost one deuterium, and none were lost from BHT-d_20._ Tajima et al. [34] had also identified this metabolite by ^13^C-NMR in rat bile. For BHT + O−2H + GSH, both BHT-d_3_ and BHT-d_20_ lost one deuterium, confirming the same mechanism as above with the *t*-butyl being hydroxylated as well. This GSH adduct was also described by Madsen et al. where they compared electrochemical and enzymatic formation of several reactive metabolites [35].

The deprotonated ion of DBP-2H + GSH did not form m/z 306 upon CID and instead had a unique fragment ion at m/z 237.1316, assigned as deprotonated DBP with the sulfur of GSH still attached. The HR-MS spectra of isotope-labeled analogs showed the loss of the three labels from BHT-d_3,_ while none were lost from BHT-d_20_ (Table 2). This supports the structure where the SG group replaces the methyl group. The formation of this metabolite is explained by a radical pathway, initiated by the decarboxylation of BHT-acid to form DBP^·^, followed by GSH trapping. Glutathione is able to scavenge radicals by its electron-donating ability, enabling it to neutralize such reactive species. This GSH adduct had not been described in previous studies.

Several glucuronide conjugates were detected, including hydroxy-BHT glucuronide (BHT + O + gluc) at 8.8 min, BHT-acid glucuronide (BHT-acid + gluc) at 8.3 min, DBP glucuronide (DBP + gluc) at 8.7 min, and BHQ glucuronide (BHQ + gluc) at 7.1 min (Figure 2 and Table 1). The MS/MS spectra of these conjugates were dominated by characteristic peaks from the glucuronide moiety, at m/z 175, m/z 113, and m/z 85, as well as the neutral loss of C_6_H_8_O_6_ (Table 1).

The only sulfate metabolite detected could be assigned as BHQ + SO_3_, eluting at 9.0 min (Figure 2). Its HRMS/MS spectra presented characteristic peaks of the sulfate conjugates at m/z 80.9645, m/z 79.9576 and m/z 221.1539 corresponding to the HSO_3_^−^ ion, the sulfonate radical ion (SO_3_**^−^**), and the neutral loss of the SO_3_ radical. This metabolite had not been reported previously.

### 2.2. Metabolism of BHA and TBHQ

BHA is an analog of BHT and has been used in different industries alone or in combination with BHT and other antioxidants. TBHQ is a metabolite of BHA through *O*-demethylation and a powerful synthetic phenolic antioxidant. BHA and TBHQ were also incubated under the same conditions studied for BHT. Table 3 summarizes LC-HRMS/MS results for BHA and all detected metabolites. The deprotonated molecules [M-H]^−^ of BHA and TBHQ were detected at *m/z* 179.1084 and *m/z* 165.0921, with retention times at 12.7 and 9.6 min, respectively. Their MS/MS spectra (Figure 1) yielded common product ions at *m/z* 149.060 and 108.021, assigned as C_9_H_9_O_2_^−^ and C_6_H_4_O_2_^−^, respectively.

Two hydroxylated BHA metabolites (BHA + O) were detected (Figure S2) at 11.4 and 11.8 min (Table 3). No significant differences were seen in their MS/MS spectra. These hydroxylated BHA isomers were attributed to the fact that BHA is a mixture of two isomers, 2-BHA (10%) and 3-BHA (90%). Armstrong et al. [36] were the first to identify 3-BHA + O by incubating pure 3-BHA (99.5%) with RLM under oxidative conditions, using ^1^H-NMR. Hydroxylated TBHQ (TBHQ + O) were also detected (Figure S3) at 8.2 min.

A dimer of BHA (C_22_H_30_O_4_) was detected at 14.4 min (Figure S2), with m/z 357.2076 (1.5 ppm). Armstrong et al. [36] also described a di-BHA metabolite [36]. BHA dimer was also found to form in rat intestine and by incubating BHA with rat intestine peroxidase, as well as horseradish peroxidase [37]. No dimer was detected for TBHQ under our conditions, which may be explained by its preference to form the quinone reactive metabolite.

Identical GSH adducts were detected for BHA and TBHQ, namely two isomers of TBHQ-2H + GSH (at 5.1 and 5.4 min), TBHQ-4H + GSH (at 6.4 min), and a di-glutathione adduct, TBHQ-4H + 2GSH (at 1.7 min) (Figures S2 and S3). The HRMS/MS spectra from these GSH adducts were dominated by characteristic fragment ions of deprotonated GSH (m/z 272, 254, 210, 179, 166, 143, and 128), with the exception of m/z 197.066 (C_10_H_13_O_2_S^−^) for the two isomers, where the sulfur atom of GSH is still bound to deprotonated TBHQ (Table 3). These GSH adducts were also detected in rat bile and urine following TBHQ administration [38]. Peters et al. [38] reported three GSH conjugates of TBHQ in vivo, including 5-(GS)-TBHQ, 6-(GS)-TBHQ, and 3,6-(GS)_2_-TBHQ by LC-MS and ^1^H-NMR. They also suggested that these conjugates could represent nephrotoxic metabolites, and may be responsible for the tumor-promoting effects of TBHQ and BHA [38]. Two novel BHA glutathione adducts have been detected here, namely BHA-2H + GSH (at 5.8 min) and BHA + O-2H + GSH (at 5.0 min) (Figure S2). The first is suggested to form via an epoxide followed by GSH addition and loss of a water molecule, while the second is likely formed via the *ortho*-quinone reactive metabolite.

Four BHA glucuronide conjugates were detected, namely TBHQ + gluc, BHA + gluc, and two isomers of BHA + O + gluc, at 3.4, 5.6, 5.7, and 6.6 min, respectively (Table 3). By comparing the MS/MS spectra of the two BHA + O + gluc isomers, the peak at 5.7 min showed a water loss at m/z 353.1259 (C_17_H_20_O_7_^−^, 2.1 ppm) proving that the oxygen is added to the *tert*-butyl group, and not in the ring as for the isomer detected at 6.6 min. These two novel metabolites have not been previously characterized. TBHQ also formed the same glucuronide conjugate as BHA (TBHQ + gluc) at 3.4 min (m/z 341.1240) with fragment ions mostly from the glucuronide moiety.

**Figure 4:**
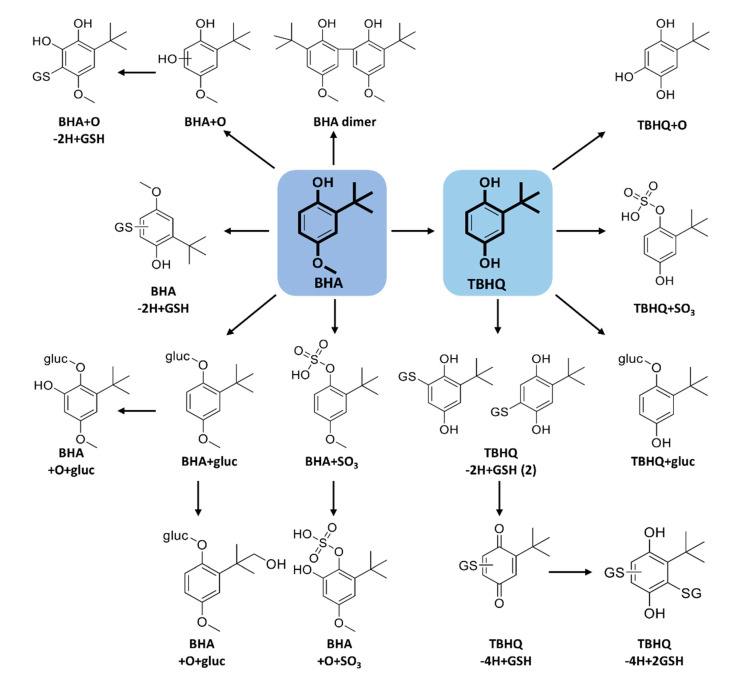
Proposed biotransformation reactions of BHA and TBHQ.

Several sulfate conjugates were detected for BHA, including two isomers corresponding to BHA + SO_3_ at 7.2 and 8.3 min. Their MS/MS spectra yielded very similar fragments suggesting that one is 3-BHA + SO_3_ and the other 2-BHA + SO_3_ (Table 3)_._ One peak corresponding to BHA + O + SO_3_ was detected at 8.4 min. A common sulfate conjugate between BHA and TBHQ (TBHQ + SO_3_) was also detected at 5.7 min. These same sulfate conjugates were previously detected in human urine using GC-MS [39]. The proposed biotransformation products of BHA and TBHQ are summarized in Figure 4.

The major metabolic pathway for BHA is reported to be via the conjugation of the free hydroxyl group with both glucuronic acid and sulfate [39]. Conning et al. [33] reported that glucuronide conjugation predominates in rat, rabbit, and human, whereas sulfation is the major phase II reaction in dog. The major TBHQ metabolites found in rat bile and urine were TBHQ-glucuronide and TBHQ-sulfate [38].

### 2.3. Metabolism of DBP and BHT Acid

DBP and BHT acid standards were used to confirm these two metabolites of BHT, as mentioned above. The oxidative metabolism for these two compounds was also studied to help support the complex metabolic pathway proposed for BHT.

Under oxidative conditions, DBP formed previously uncharacterized oxidative metabolites, including a major metabolite hydroxylated of the *t*-butyl group (DBP + O), as well as two di-hydroxylated forms and a carboxylic acid metabolite (DBP + 2O−2H). Figure S1 shows the extracted ion chromatograms for DBP and BHT-acid oxidative metabolites, as well as a proposed scheme for their formation, supported by high-resolution MS/MS spectra for each metabolite.

The BHT-acid standard confirmed this BHT metabolite and helped distinguish it from the novel (BHT-aldehyde) + O. BHT-acid incubations showed that the formation of DBP and BHQ was non-enzymatic, and allowed a novel hydroxylated (BHT-acid + O) metabolite to be characterized (Figure S1).

By compiling the results from in vitro biotransformations of BHT, BHA, TBHQ, DBP, and BHT-acid, comprehensive schemes of the many metabolic transformations for these small synthetic antioxidants have been proposed. Using an untargeted high-resolution tandem mass spectrometry approach to decipher the metabolism of analogous compounds, while incorporating isotope labeling, proved to be a powerful method to elucidate structures of all detected metabolites.

## 3. Methods

### 3.1. Chemicals

Butylated hydroxytoluene [BHT, 2,6-di-tert-butyl-4-methylphenol,], butylated hydroxyanisole [BHA, 2(3)-tert-butyl-4-methoxyphenol (90%/10%)], tert-butylhydroquinone [TBHQ, 2-tert-butylbenzene-1,4-diol], 2,6-di-tert-butylphenol [DBP, 2,6-bis(tert-butyl)phenol], BHT acid [3,5-Di-tert-butyl-4-hydroxybenzoic acid], glutathione (GSH), uridine 5′-diphosphoglucuronic acid (UDPGA), 3′-phosphoadenosine-5′-phosphosulfate (PAPS), nicotinamide adenine dinucleotide phosphate (NADP ^+^ ), glucose-6-phosphate, MgCl_2_ and glucose-6-phosphate dehydrogenase, as well as HPLC-grade acetonitrile (ACN), methanol and formic acid were all purchased from Sigma-Aldrich (Oakville, ON, Canada). BHT-d_3_ [2,6-di-tert-butyl-4-methyl-d3-phenol] and BHT-d_21_ [2,6-di-(tert-butyl-d_9_)-4-methylphenol-3,5-d_2_,OD] were purchased from CDN Isotopes (Pointe-Claire, QC, Canada). BHT-d_20_ was created by heating the BHT-d_21_ for 2 h at 60 °C. Human and rat liver microsomes (HLM and RLM) and human and rat liver S9 (HS9 and RS9) fractions were purchased from Corning (Corning, NY, USA). Ultrapure water was from a Millipore Synergy UV system (Billerica, MA, USA).

### 3.2. In Vitro Incubations

#### 3.2.1. Oxidative Metabolites and GSH Adducts

BHT and analogs were incubated at 20 µM with human and rat liver microsomes (1 mg/mL protein) containing 5 mM GSH and a NADPH-regenerating system (5 mM MgCl_2_, 0.5 mM NADP ^+^, 10 mM glucose-6-phosphate and 2 units/mL glucose-6-phosphate dehydrogenase) at 37 °C for 1 h in 100 mM phosphate buffer, pH 7.4. Control samples were prepared without NADPH regenerating system and/or without GSH. Adding an equal volume of cold acetonitrile quenched the reaction. Incubation mixtures were centrifuged for 8 min at 14,000 rpm, at 4 °C. The supernatants were diluted (1:1) in water prior to LC-MS/MS analysis.

#### 3.2.2. Phase II Metabolism—Glucuronidation and Sulfation

To study the glucuronide conjugates, all compounds (20 µM) were incubated with HLM and RLM (1 mg/mL protein), NADPH-regenerating system (as above), and 5 mM UDPGA at 37 °C for 1h. Each compound (20 µM) was also incubated with human and rat S9 fractions (2 mg/mL) in phosphate buffer, containing 1 mM of PAPS with the NADPH-regenerating system, to study the formation of sulfates conjugates. All samples were incubated at 37 °C for 1h, quenched, and centrifuged as above. Supernatants were diluted as above and subjected to LC-MS/MS analysis.

### 3.3. LC-HRMS/MS Analysis and Data Processing

LC-MS/MS analyses were performed using a Shimadzu Nexera HPLC coupled to a Sciex 5600 TripleTOF^®^ (quadrupole-time-of-flight) system (Concord, ON, Canada), in negative electrospray mode.

Chromatographic separation was performed using a Phenomenex Kinetex biphenyl (100 × 2.1 mm, 2.6 µm) column, using mobile phases of 5 mM ammonium acetate in water and 100% ACN, at 0.25 mL/min and a column temperature of 40 °C. The injection volume was 25 µL. The HPLC gradient was as follows: 5% B held for 0.5 min, linearly increased to 30% at 8 min, up to 50% at 12 min, and 90% at 13 min, held for an additional 2 min.

Ion source parameters were as follows: ionization voltage at 5000 V, curtain gas of 35 psi, drying and nebulizer gases each at 50 psi, source temperature of 450 °C, and declustering potential of 60 V. TOF-MS spectra were acquired (with 250 ms accumulation time), followed by MS/MS in information-dependent acquisition (IDA) mode on the 5 most intense ions using dynamic background subtraction (175 ms each). Nitrogen was used as collision gas and collision energy was 30 ± 10 V. Metabolites that did not have high-quality MS/MS in IDA mode, targeted MS/MS mode was used in a second injection.

MetabolitePilot 2.0 (Sciex) software was employed to screen samples for potential metabolites using a set of known biotransformations, including oxidative reactions, GSH, glucuronide, and sulfate conjugates. PeakView 2.2 and MasterView 1.1 (Sciex) were also used for processing LC-MS/MS data to confirm and expand the list of features based on mass accuracy, isotope pattern, and MS/MS analysis.

## 4. Conclusions

The metabolism of BHT and several analogs were investigated in vitro using human and rat liver microsomes and S9 fractions. Many oxidative metabolites, GSH adducts, glucuronide, and sulfate conjugates were detected with excellent mass accuracy, some of which had not been previously reported. Structures of biotransformation products were elucidated by HRMS/MS data and supported using isotope-labeled analogs. These results have enabled many biotransformation products to be determined, which are potentially involved in the toxicity of these compounds. Knowledge of all these possible metabolites would be useful in assessing environmental exposure to these compounds.

## Figures and Tables

**Table 1:** Summary of MS/MS data for BHT and metabolites in negative ion mode.

Metabolite	Formula	*m/z* [M-H]^−^	ppm	RT (min)	Selected MS/MS Fragments *
**BHT**	C_15_H_24_O	219.1760	2.7	14.3	203.1439
**BHT + O (1)**	C_15_H_24_O_2_	235.1708	2.1	13.4	**217.1598**, **160.0895**, 145.0657
**BHT + O (2)**	C_15_H_24_O_2_	235.1705	0.8	14.1	217.1597, **205.1597**
**BHT + 2O**	C_15_H_24_O_3_	251.1660	2.9	11.9	**233.1547**, 221.1557, **203.1444**, 160.0893, 146.0746
**BHT−aldehyde**	C_15_H_22_O_2_	233.1552	2.2	14.0	217.1234
**BHT−acid**	C_15_H_22_O_3_	249.1501	2.2	9.8	**205.1592**
**BHT−aldehyde + O**	C_15_H_22_O_3_	249.1503	3.0	13.5	231.1400, **219.1399**
**DBP**	C_14_H_22_O	205.1600	1.2	14.2	**189.1289**
**BHQ**	C_14_H_22_O_2_	221.1549	0.9	8.3	164.0848, 149.0601
**BHT−2H + GSH**	C_25_H_39_N_3_O_7_S	524.2436	0.1	8.5	**306.0772**, 272.0892, 254.0788, 160.0078, **143.0463**, 128.0353
**BHT + O-2H + GSH**	C_25_H_39_N_3_O_8_S	540.2381	−0.7	7.6	**306.0758**, 288.0646, 272.0882, 254.0778, 160.0078, **143.0465**, 128.0348
**DBP-2H + GSH**	C_24_H_37_N_3_O_7_S	510.2287	1.5	8.2	492.2179, 272.0886, 254.0793, 237.1316, 143.0462, 128.0353
**DBP + gluc**	C_20_H_30_O_7_	381.1924	1.4	8.7	**205.1595**, 175.0247, **113.0240**, 85.0291
**BHT + O + gluc**	C_21_H_32_O_8_	411.2026	0.5	8.8	193.0360, 131.0341, 113.0244, 85.0289
**BHT−acid + gluc**	C_21_H_30_O_9_	425.1820	0.9	8.3	249.1491, **193.0354**, **175.0247**, **131.0344**, **113.0239**, **72.9929**
**BHQ + gluc**	C_20_H_30_O_8_	397.1864	−1.0	7.1	221.1537, 175.0135, 113.0242, 85.0290
**BHQ + SO_3_**	C_14_H_22_O_5_S	301.1117	0.8	9.0	**221.1539**, 164.0844, 80.9645, **79.9576**

* fragment ions of >20% intensity relative to base peak are listed here in bold, base peak in each spectrum is underlined.

**Table 2:** High-resolution mass spectrometry data using isotope-labeled BHT analogs.

Metabolite	BHT-d_3_ Formula *m/z* (ppm)	BHT-d_20_ Formula *m/z* (ppm)	Comments
BHT + O (1)	C_15_D_2_H_22_O_2_ 237.1834 (2.1)	C_15_D_20_H_4_O_2_ 255.2967 (3.2)	O added on *p*-CH_3_
BHT + O (2)	C_15_D_3_H_21_O_2_ 238.1893 (0.7)	C_15_D_19_H_4_O_2_ 254.2903 (2.6)	O added on *t*-butyl
BHT + 2O	C_15_D_2_H_22_O_3_ 238.1893 (0.7)	C_15_D_19_H_4_O_3_ 270.2855 (3.6)	O added on *p*-CH_3_ and *t*-butyl
BHT−aldehyde	C_15_H_22_O_2_ 233.1553 (2.5)	C_15_D_20_H_2_O_2_ 253.2811 (3.4)	Aldehyde in *para* position
BHT−acid	C_15_H_22_O_3_ 249.1505 (3.5)	C_15_D_20_H_2_O_3_ 269.2757 (2)	Carboxylic acid in *para* position
BHT−aldehyde + O	C_15_H_22_O_3_ 249.1504 (3.1)	C_15_D_19_H_2_O_3_ 268.2689 (0.1)	CHO on *p*-methyl, OH on *t*-butyl
DBP	C_14_H_22_O 205.1607 (4.4)	C_14_D_20_H_2_O 225.2861 (3.4)	DBP structure
BHQ	C_14_H_22_O_2_ 221.1548 (0.4)	C_14_D_20_H_2_O_2_ 241.2797 (−2.2)	O added to DBP in *para* position
BHT−2H + GSH	C_25_D_2_H_37_N_3_O_7_S 526.2560 (−0.2)	C_25_D_20_H_19_N_3_O_7_S 544.3691 (−0.1)	SG group added to *p*-CH_3_
BHT + O−2H + GSH	C_25_D_2_H_37_N_3_O_8_S 542.2508 (−0.4)	C_25_D_19_H_20_N_3_O_8_S 559.3588 (1.8)	SG added to *p*-CH_3_ and O on *t*-butyl
DBP−2H + GSH	C_24_H_37_N_3_O_7_S 510.2274 (−1.1)	C_24_D_20_H_17_N_3_O_7_S 530.3541 (1.2)	SG added to ring in *para* position
DBP + gluc	C_20_H_30_O_7_ 381.1924 (1.4)	C_20_D_20_H_10_O_7_ 401.3184 (2.5)	Glucuronide on hydroxyl of DBP
BHT + O + gluc	C_21_D_3_H_29_O_8_ 414.2222 (2.2)	C_21_D_19_H_13_O_8_ 430.3218 (0.2)	Glucuronide on O added on *t*-butyl
BHT−acid + gluc	C_21_H_30_O_9_ 425.1821 (0.9)	C_21_D_20_H_10_O_9_ 445.3068 (−1.0)	Glucuronide on carboxylic acid
BHQ + gluc	C_20_H_30_O_8_ 397.1877 (2.3)	C_20_D_20_H_10_O_8_ 417.3120 (0.8)	Glucuronide added to *para*-OH
BHQ + SO_3_	C_14_H_22_O_5_S 301.1116 (0.5)	C_14_D_20_H_10_O_9_ 321.2369 (−0.4)	SO_3_ attached to BHQ

**Table 3:** Summary of MS/MS data for BHA metabolites in negative ion mode.

Metabolite	Formula	*m/z* [M-H]^−^	ppm	RT (min)	Selected MS/MS Fragments *
**BHA**	C_11_H_16_O_2_	179.1084	3.6	12.7	**164.0838**, **149.0604**, 121.0656, 108.0213
**BHA + O (1)**	C_11_H_16_O_3_	195.1027	2.3	11.8	**180.0795**, **165.0560**, 137.0609
**BHA + O (2)**	C_11_H_16_O_3_	195.1031	2.4	11.4	**180.0784**, **165.0552**
**BHA dimer**	C_22_H_30_O_4_	357.2076	1.5	14.4	**342.1831**, **327.1608**
**BHA-2H + GSH**	C_21_H_31_N_3_O_8_S	484.1760	0.2	5.8	**272.0898**, **254.0791**, **211.0810**, 210.0884, 179.0456, **143.0461**, **128.0345**
**BHA + O-2H + GSH**	C_21_H_31_N_3_O_9_S	500.1709	0.3	5.0	**306.0759**, 272.0864, 254.0750, 210.0892, 179.0450, 160.0056, 143.0470, 128.0371
**TBHQ-2H + GSH (1)**	C_20_H_29_N_3_O_8_S	470.1602	−0.1	5.1	**272.0924**, **254.0822**, **210.0916**, **197.0660**, **179.0479**, 146.0468, **143.0471**, **128.0358**
**TBHQ-2H + GSH (2)**	C_20_H_29_N_3_O_8_S	470.1675	2.3	5.4	**272.0925**, **254.0821**, 210.0911, **197.0662**, 185.0586, 179.0476, 166.0998, 146.0462, **143.0466**, **128.0358**
**TBHQ-4H + GSH**	C_20_H_27_N_3_O_8_S	468.1446	0.6	6.4	339.1025, **272.0930**, 254.0811, 210.0896, **197.0657**, **195.0503**, **192.1049**, 179.0465, 146.0442, **143.0466**, **128.0353**
**TBHQ-4H + 2GSH**	C_30_H_44_N_6_O_14_S_2_	775.2288	0.6	1.7	646.1861, **502.1334**, 468.1445, 306.0769, 272.0890, 254.0790, 229.0358, 195.0488, 143.0463
**BHA + gluc**	C_17_H_24_O_8_	355.1401	0.9	5.6	337.1300, 179.1082, **175.0252**, **164.0845**, 117.0196, **113.0243**, 85.0296, 75.0090, 59.0139
**BHA + O + gluc (1)**	C_17_H_24_O_9_	371.1351	1	5.7	353.1259, 265.1083, **195.1035**, **180.0800**, 175.0254, 113.0245, 85.0293, 75.0080, 59.0135
**BHA + O + gluc (2)**	C_17_H_24_O_9_	371.1357	2.6	6.6	**195.1027**, **180.0792**, 175.0242, 113.0237, 85.0287
**TBHQ + gluc**	C_16_H_22_O_8_	341.1240	−0.4	3.4	175.0253, **165.0920**, **113.0247**, 85.0295, 59.0137
**BHA + SO_3_ (1)**	C_11_H_16_O_5_S	259.0647	4.7	8.3	244.0405, 179.1070, **164.0842**, 149.0606, **70.9571**
**BHA + SO_3_ (2)**	C_11_H_16_O_5_S	259.0645	0.4	7.2	239.0718, **179.1077**, **164.0844**, 149.0635, **79.9571**
**BHA + O + SO_3_**	C_11_H_16_O_6_S	275.0597	0.8	8.4	**195.1032**,**180.0794**, 165.0559, 79.9574
**TBHQ + SO_3_**	C_10_H_14_O_5_S	245.0489	1.4	5.7	165.0923, 149.0607, 108.0213, 80.9651, 79.9572

* fragment ions of >20% intensity relative to base peak are listed here in bold, base peak in each spectrum in underlined.

## Supplementary Materials

The following are available online at https://www.mdpi.com/1422-0067/21/24/9525/s1, Figure S1: Extracted ion chromatograms of oxidative metabolites formed when BHT-acid (A) and DBP (B) were incubated with HLM and NADPH. (C) Proposed biotransformation reactions of DBP and BHT-acid; Figure S2: Extracted ion chromatograms of BHA metabolites formed in HLM with added (A) NADPH, (B) NADPH and GSH, (C) NADPH and UDPGA, and (D) human S9 fraction with NADPH and PAPS. Figure S3: Extracted ion chromatograms of TBHQ metabolites formed in (A) HLM with NADPH; (B) HLM with NADPH and GSH; (C) HLM with NADPH and UDPGA; (D) human S9 fraction with NADPH and PAPS.
