# Supplementary material for: Identification of In Vitro Metabolites of Synthetic Phenolic Antioxidants BHT, BHA, and TBHQ by LC-HRMS/MS
Source: Int J Mol Sci. 2020 Dec 15;21(24):9525. doi: 10.3390/ijms21249525 (PMC7765162; doi:10.3390/ijms21249525)
Supplement: Supplementary file 1 [file ijms-21-09525-s001.pdf]

Supplemental information

Identification of *in vitro* metabolites of synthetic phenolic antioxidants BHT, BHA and TBHQ by LC-HRMS/MS

Ons Ousji and Lekha Sleno*

*Corresponding author: sleno.lekha@uqam.ca

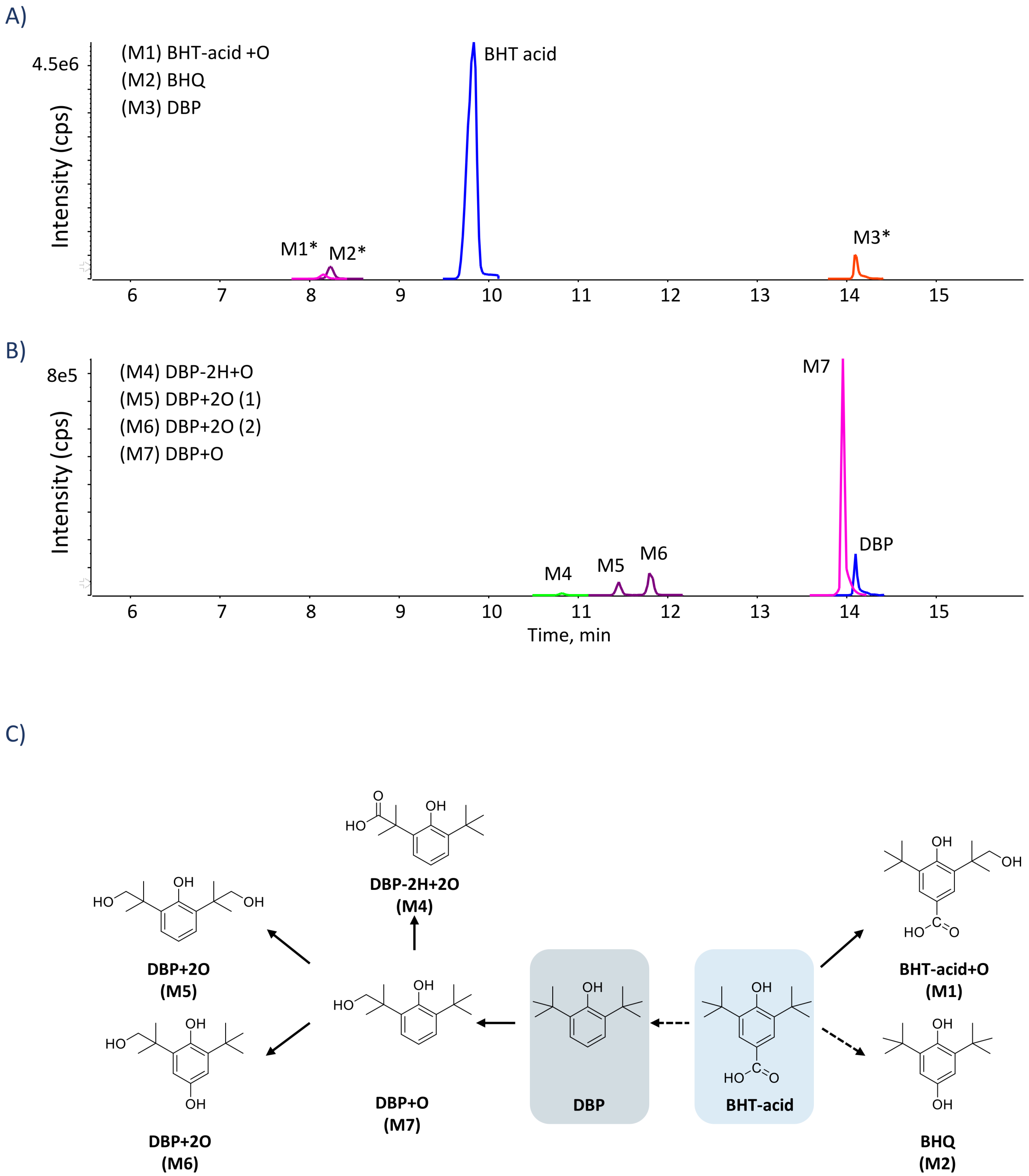

Figure S1. Extracted ion chromatograms of oxidative metabolites formed when BHT-acid (A) and DBP (B) were incubated with HLM and NADPH; Peaks with asterisk (*) were increased by 5x. (C) Proposed biotransformation reactions of DBP and BHT-acid; dotted arrows refer to non-enzymatic reactions (peaks found in control without incubation time, quenched at 0 min)

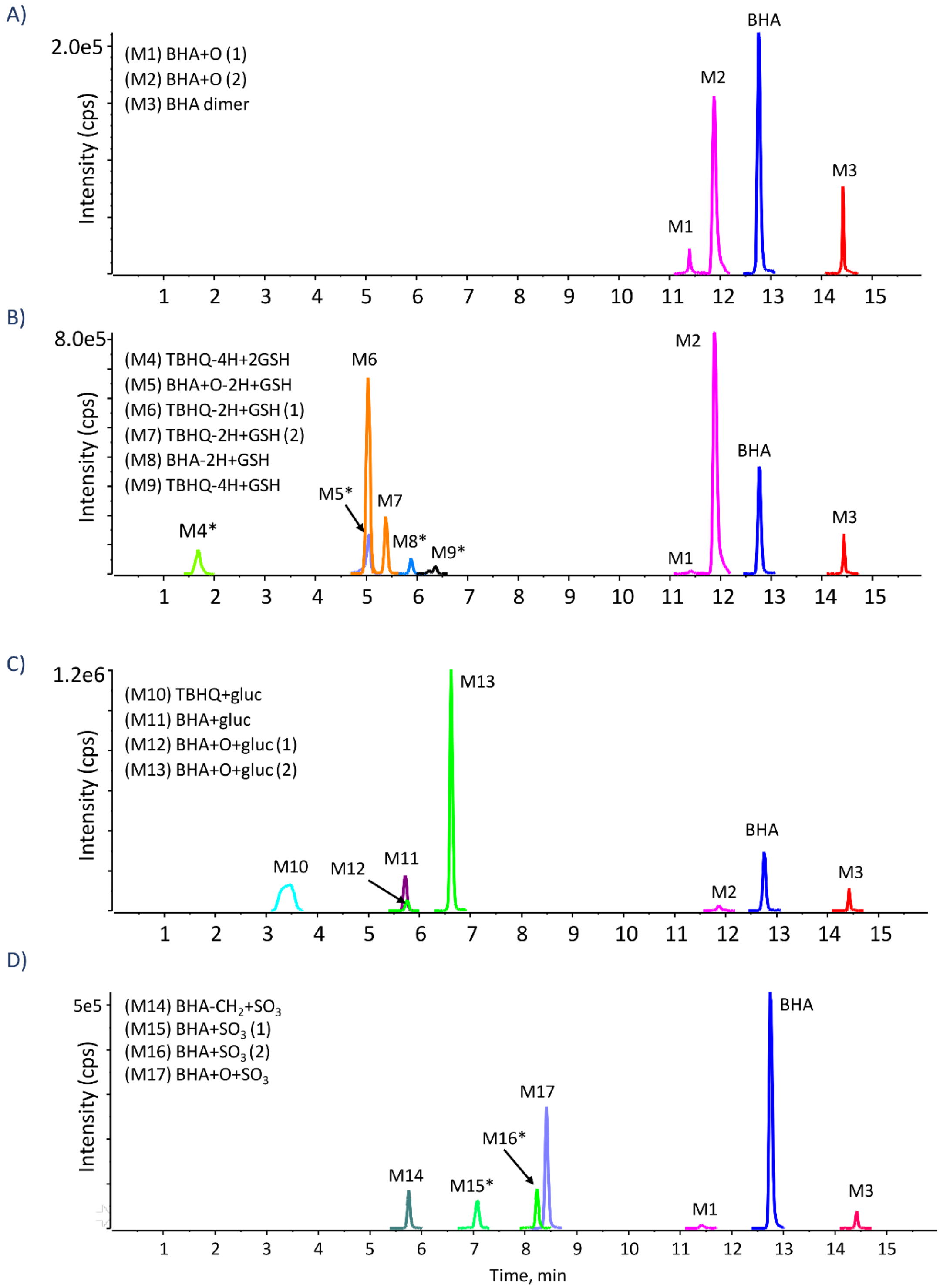

Figure S2. Extracted ion chromatograms of BHA metabolites formed in HLM with added (A) NADPH, (B) NADPH and GSH, (C) NADPH and UDPGA, and (D) Human S9 fraction with NADPH and PAPS. Peaks with asterisk (*) were increased by 10x.

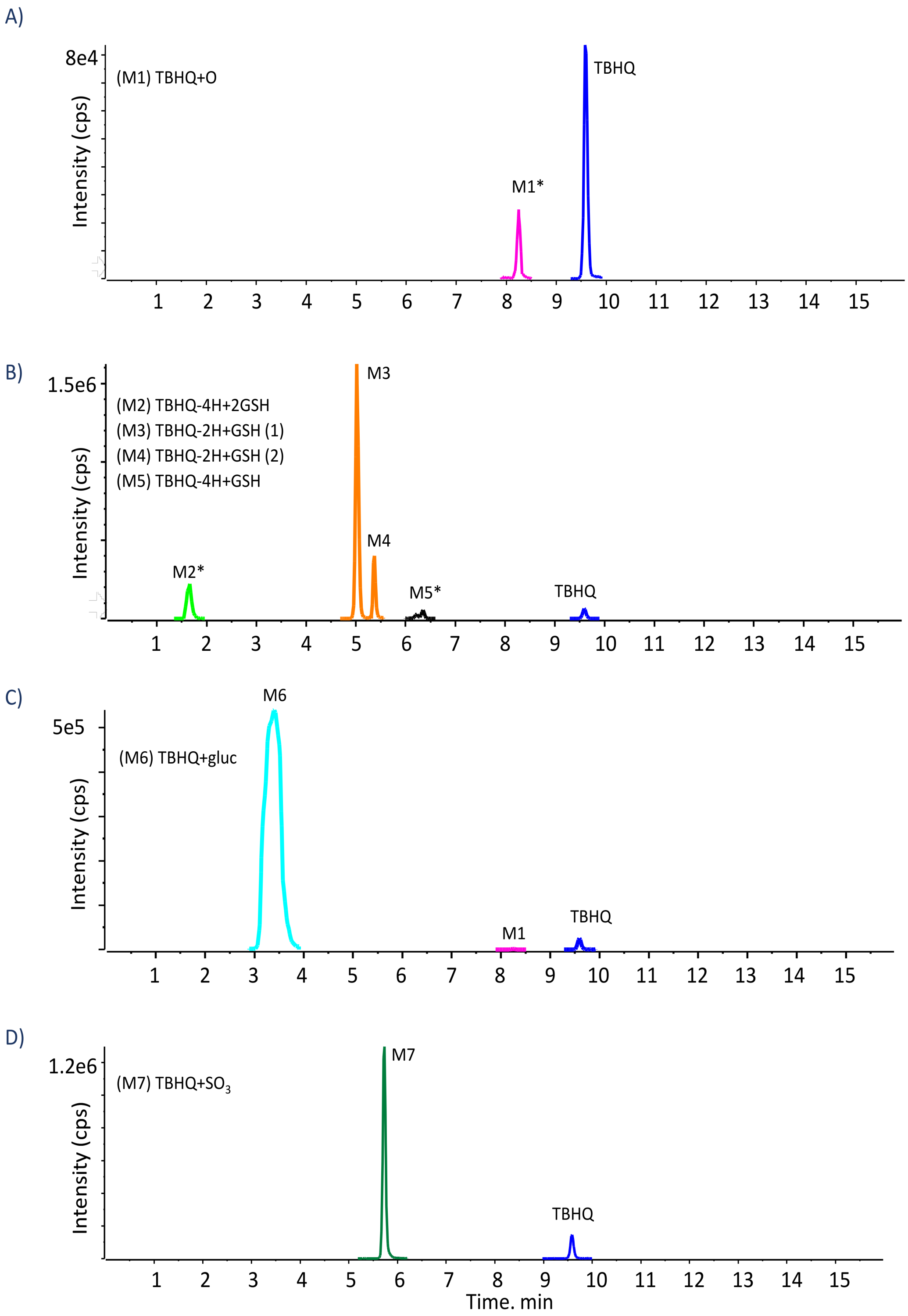

Figure S3. Extracted ion chromatograms of TBHQ metabolites formed in (A) HLM with NADPH; (B) HLM with NADPH and GSH; (C) HLM with NADPH and UDPGA; (D) Human S9 fraction with NADPH and PAPS; Peaks with asterisk (*) were increased by 10x.
